# Profiling and analysis of chemical compounds using pointwise mutual information

**DOI:** 10.1186/s13321-020-00483-y

**Published:** 2021-01-10

**Authors:** I. Čmelo, M. Voršilák, D. Svozil

**Affiliations:** 1 CZ-OPENSCREEN National Infrastructure for Chemical Biology, Department of Informatics and Chemistry, Faculty of Chemical Technology, University of Chemistry and Technology Prague, Technická 5, 166 28 Prague, Czech Republic; 2 CZ-OPENSCREEN National Infrastructure for Chemical Biology, Institute of Molecular Genetics of the ASCR v. v. i., Vídeňská 1083, 142 20 Prague 4, Czech Republic

**Keywords:** Hashed fingerprint, Structural key, Information theory, Pointwise mutual information, Synthetic accessibility

## Abstract

Pointwise mutual information (PMI) is a measure of association used in information theory. In this paper, PMI is used to characterize several publicly available databases (DrugBank, ChEMBL, PubChem and ZINC) in terms of association strength between compound structural features resulting in database PMI interrelation profiles. As structural features, substructure fragments obtained by coding individual compounds as MACCS, PubChemKey and ECFP fingerprints are used. The analysis of publicly available databases reveals, in accord with other studies, unusual properties of DrugBank compounds which further confirms the validity of PMI profiling approach. Z-standardized relative feature tightness (ZRFT), a PMI-derived measure that quantifies how well the given compound’s feature combinations fit these in a particular compound set, is applied for the analysis of compound synthetic accessibility (SA), as well as for the classification of compounds as easy (ES) and hard (HS) to synthesize. ZRFT value distributions are compared with these of SYBA and SAScore. The analysis of ZRFT values of structurally complex compounds in the SAVI database reveals oligopeptide structures that are mispredicted by SAScore as HS, while correctly predicted by ZRFT and SYBA as ES. Compared to SAScore, SYBA and random forest, ZRFT predictions are less accurate, though by a narrow margin (*Acc*_*ZRFT*_ = 94.5%, *Acc*_*SYBA*_ = 98.8%, *Acc*_*SAScore*_ = 99.0%, *Acc*_*RF*_ = 97.3%). However, ZRFT ability to distinguish between ES and HS compounds is surprisingly high considering that while SYBA, SAScore and random forest are dedicated SA models, ZRFT is a generic measurement that merely quantifies the strength of interrelations between structural feature pairs. The results presented in the current work indicate that structural feature co-occurrence, quantified by PMI or ZRFT, contains a significant amount of information relevant to physico-chemical properties of organic compounds.

## Introduction

Information theory is a mathematical approach for the quantification, storage and communication of information. Information theory concepts, such as Shannon entropy [1] or mutual information (MI) [2], are used across a wide variety of scientific areas. Due to the generic nature of information theory, sometimes even very distant scientific fields independently develop methodologies that are built upon the same underlying information theory framework. In one such framework, MI is used to profile and compare objects based on the interrelations between their features. MI is commonly used in linguistics to identify unusual word combinations [3] with the aim to estimate text complexity [4]. In bioinformatics, gene coinheritance among different organisms, expressed by MI, was profiled to elucidate functional linkages among proteins [5]. In medicinal sciences, MI was applied to profile relations between stressors, health conditions, genes and other factors in order to build comorbidity charts useful for disease study and preventive medicine [6–8].

In cheminformatics, the use of information theory concepts is widespread [9, 10]. Shannon entropy was applied, for example, to design and evaluate molecular descriptors [11, 12] and fingerprints [13], to determine the information content of chemical structures based on their topology and symmetry [14], to create the aggregate fingerprints of whole chemical databases [15] or to evaluate the significance of individual fingerprint bits in order to improve similarity search methodologies [16]. MI was applied to improve feature selection in similarity search [17] and QSAR [18, 19] and to improve performance of topological molecular descriptors in the modeling of the physico‐chemical properties of 2-furylethylene derivatives [20]. However, a more straightforward MI application, the comparison of compound sets based on interrelations between their structural features, was not reported so far. In this paper, we demonstrate the use of pointwise mutual information (PMI) for the profiling of structural feature interrelations within several publicly available chemical databases (DrugBank [21], ChEMBL [22, 23], PubChem [24] and ZINC15 [25]) using PubChem [26] and MDL MACCS [27] structure keys, as well as extended connectivity fingerprints (ECFP) [28]. Z-standardized relative feature tightness (ZRFT), a PMI-based measure that quantifies how the given compound fits into the particular compound set, is postulated and its utility is demonstrated in the analysis of compound synthetic accessibility (SA), as well as in the classification of compounds as easy (ES) and hard (HS) to synthesize.

## Methods

### Methodology of feature interrelation profiling

In linguistics, PMI is used to expresses the extent to which the observed frequency of the co-occurrence of two different words differs from what would be expected if they were independent [29]. PMI is the measure of the strength of the association between words *x* and *y* and, for a given corpus, it is calculated using the number of times the word pair (*x*, *y*) is observed in one sentence versus the number of times words *x* and *y* are observed separately. The concept of PMI can be easily adopted for the analysis of the interrelations between structural features (i.e., words) within individual molecules (i.e., sentences) from a compound set (i.e., a corpus). In this work, two types of structural features are employed: dictionary-based and hashed structural fragments [30–32]. Dictionary-based fragments are used to convert a compound into a binary fingerprint called “a structure key”. Though fragment dictionaries are constructed from fragments perceived as most relevant to the intended purpose, some important fragments may be omitted. To circumvent this aspect of explicit fragment selection, hashed fingerprints were developed. They are formed by fitting all fragments present in the molecule up to a defined size into the bit-string of the defined length. In the present work, PubChem [26] and MDL MACCS [27] structure keys and ECFP4 and ECFP6 [28] hashed fingerprints are used to decompose molecules into structural features. Structural features/fragments will be, in the following text, referred to simply as features.

Profiling feature interrelations requires to retain information on how many times each feature pair appears in the compound set *S*. This information is stored in the co-occurrence relation matrix (CORM). If each molecule in the compound set *S* is encoded by the feature vector *k*, CORM is calculated as the sum of the outer products of all feature vectors *k*:

1 $$CORM\left( S \right) = \mathop \sum \limits_{o~ = ~1}^{\left| S \right|} {k_o} \otimes {k_o} = ~\mathop \sum \limits_{o~ = ~1}^{\left| S \right|} {k_o}k_o^T$$

where *|S|* is the number of molecules in the compound set *S*. CORM is a symmetrical square matrix of nonnegative integers with dimensions equaling to the number of features, i.e. to the length of the feature vector *k*.

The division of co-occurrence counts in CORM by compound set size *|S|* leads to the co-occurrence probability relation matrix (COPRM):

2 $$COPRM(S) = \frac{CORM(S)}{|S|}$$

On its diagonal, COPRM contains probabilities with which individual features are observed in the compound set *S*. Its off-diagonal elements contain probabilities of the occurrence of feature pairs in the compound set *S*.

The strength of the interrelation between two features *x* and *y* can be inferred using pointwise mutual information (PMI):

3 $$PMI={\mathrm{log}}_{2}\frac{p(x,y)}{p(x)p(y)}$$

PMI quantifies the divergence between feature pair co-occurrence probability *p(x, y)* and individual occurrence probabilities *p(x)* and *p(y).* Positive PMI indicates the enrichment of feature co-occurrences compared to their separate occurrences, e.g., PMI of 1 means that both features appear together (i.e., in one compound) twice as often as they appear separately (i.e., in two different compounds). PMI equaling to 0 means that two features appear together about as often as they appear separately. Negative PMI indicates negative interrelation between a pair of features, e.g., a feature pair with PMI of -1 appears only half as often as could be expected from their individual occurrence probabilities.

From COPRM, a pointwise mutual information relation matrix (PMIRM) containing PMI values for all possible feature pairs can be constructed. Its individual elements *PMIRM*(*S*)_*i*,*j*_ are given as:

4 $$PMIRM{\left(S\right)}_{i,j} = {log}_{2} \frac{COPRM{\left(S\right)}_{i,j}}{COPRM{\left(S\right)}_{i,i} COPRM{\left(S\right)}_{j,j}}$$

**Fig. 1:**
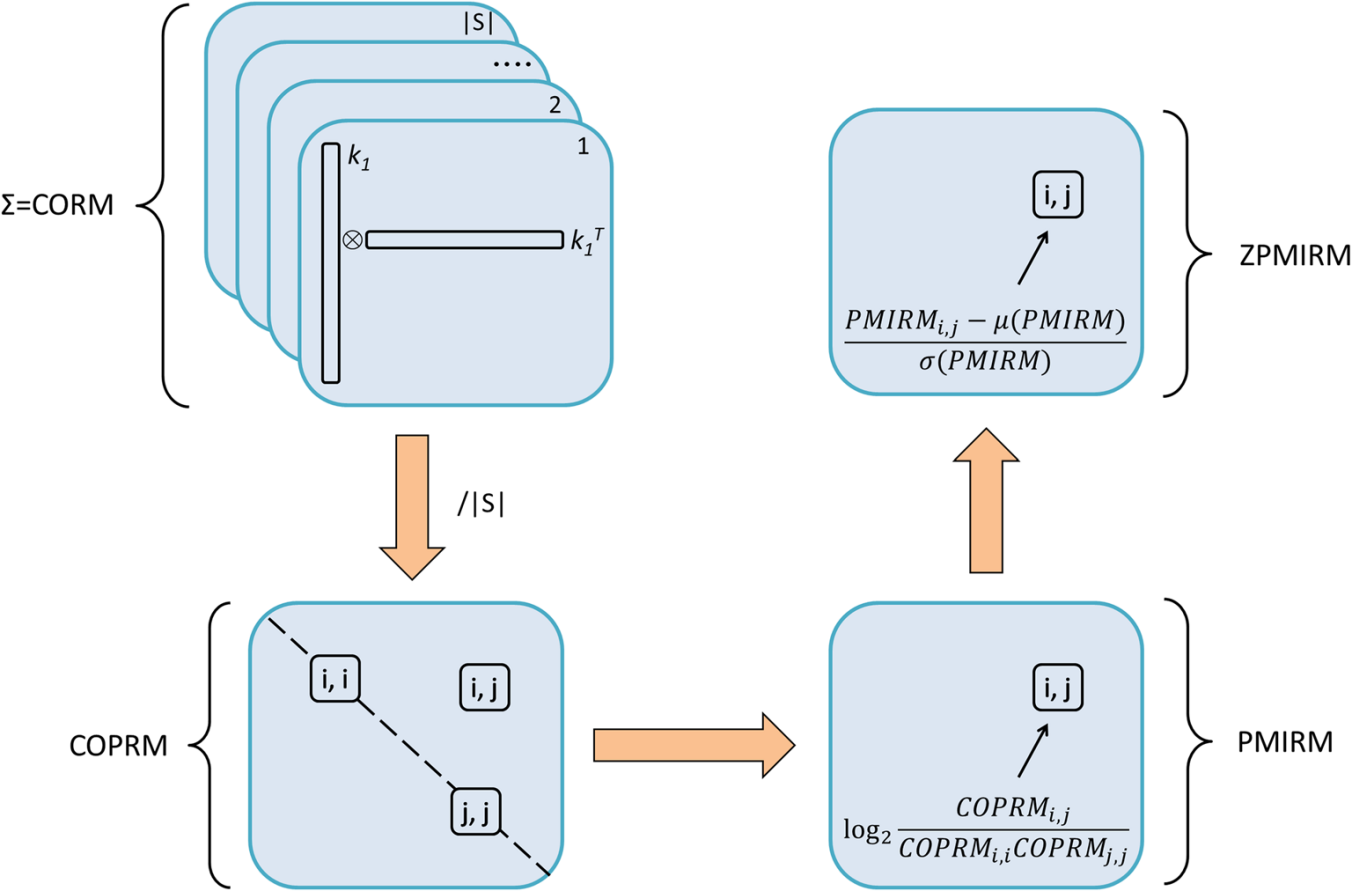
Relation matrices. Co-occurrence relation matrix (CORM) is the sum of the outer products of all feature vectors *k*. Individual elements of co-occurrence probability relation matrix (COPRM) are calculated by dividing corresponding CORM elements by the number of feature vectors (i.e., by the size of the compound set *S*) |*S*|. Pointwise mutual information relation matrix (PMIRM) consists of PMI between all feature pairs *i* and *j*. Z-standardized pointwise mutual information relation matrix (ZPMIRM) is obtained from PMIRM by converting its elements into Z-scores

PMIRM diagonal contains zeros and feature pairs involving features that are never observed in the compound set *S* have undefined PMI. PMIRM constitutes the interrelation profile of the compound set *S*. PMIRM interrelation profile is intrinsically affected by the choice of features. For example, overlapping structural features can interact in a complementary manner which leads to the shift of PMI distribution towards positive values. These shifts can be, if desired, corrected by normalizing PMI values into Z-scores (ZPMI) leading to the Z-standardized pointwise mutual information relation matrix (ZPMIRM):

5 $$ZPMIRM{\left(S\right)}_{i,j} =\frac{PMIRM{\left(S\right)}_{i,j} - \mu (PMIRM(S))}{\sigma (PMIRM(S))}$$

where *μ* is the mean and *σ* is the standard deviation of all values in PMIRM. The construction of relation matrices (RMs) CORM, COPRM, PMIRM and ZPMIRM is summarized in Fig. 1.

Apart from the analysis of interrelations within the compound set *S*, PMI methodology also enables to measure how tightly the query compound set *S* matches the reference compound set *S’* meaning how similar are, on average, the query and reference compound sets in terms of feature pair co-occurrence probabilities. This is quantified by the relative feature tightness (RFT):

6 $$RFT=\upmu \left(COPRM\left(S\right)\times PMIRM\left(S{^{\prime}}\right)\right)=\upmu \left(\frac{{\sum }_{o = 1}^{|S|}{k}_{o}{k}_{o}^{T}}{|S|}\times PMIRM(S{^{\prime}})\right)$$

where COPRM(*S*) is the co-occurrence probability relation matrix (Eq. 2) of the query compound set *S*, PMIRM(*S’*) is the pointwise mutual information relation matrix (Eq. 4) of the reference compound S’ and *μ* is the mean of all values in the $COPRM\left(S\right)\times PMIRM\left(S{^{\prime}}\right)$ matrix. Based on the choice of *S* and *S’*, three different cases can occur:

1. The query compound set *S* consists of only one compound, the reference compound set *S’* consists of several compounds. In this case, RFT measures how well the feature combinations of a compound S fit these within the reference compound set *S’*.
2. Both *S* and *S’* compound sets consist of several compounds. In this case, RFT measures how close are feature interrelations within compounds from the query compound set *S* to feature interrelations within the reference compound set *S’*.
3. The reference compound set *S’* is the same as the query compound set *S*, i.e., *S* = *S’*. In this case, RFT measures the “inner tightness” of the compound set *S*, i.e. how strong are the feature interrelations within the compound set *S*.

Generally, the higher RFT is, the more similar are the compound sets *S* and *S’* in terms of feature co-occurrences. If ZPMIRM is used instead of PMIRM in Eq. 6, a Z-standardized relative feature tightness (ZRFT) is obtained:

7 $$ZRFT=\upmu \left(COPRM\left(S\right)\times ZPMIRM\left(S{^{\prime}}\right)\right)=\upmu \left(\frac{{\sum }_{o = 1}^{|S|}{k}_{o}{k}_{o}^{T}}{|S|}\times ZPMIRM(S{^{\prime}})\right)$$

ZRFT is interpreted much like RFT with the added convenience of standardization: chemical structures containing predominantly feature pairs that are rated above average within the reference interrelation profile will receive positive ZRFT values and vice versa. However, it must be stressed that neither RFT, nor ZRFT can be considered as metrics because they are not symmetric: *RFT/ZRFT(A, B)* is unlikely to be the same as *RFT/ZRFT(B, A)*.

### Applications of feature interrelation profiling

The utility of feature interrelation profiling is demonstrated for chemical database and synthetic accessibility analysis.

#### Chemical database analysis

**Fig. 2:**
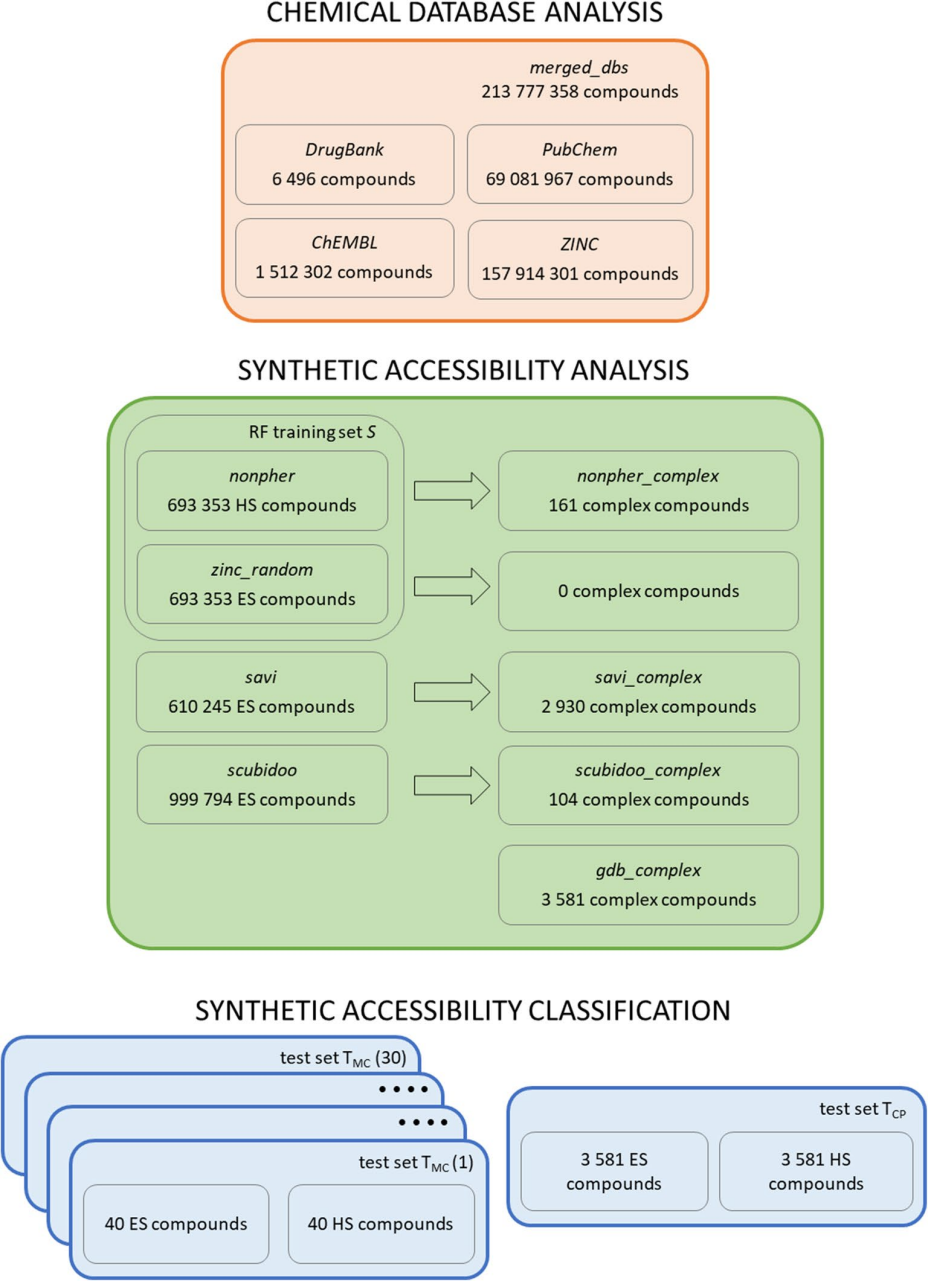
List of compound sets. Synthetic accessibility interrelation patterns are analyzed for one set of HS compounds (*nonpher* compound set) and three sets of ES compounds (*zinc_random*, *savi* and *scubidoo* compound sets). Extremely complex compounds in these data sets (*_complex* compound subsets) are also considered to be HS. *zinc_random* compound set does not contain any excessively complex compound. *nonpher* and *zinc_random* compound sets are augmented into the training set *S* used to train the RF classifier

In this application, the DrugBank 5.0.3 [21], ChEMBL22 [22, 23], PubChem (downloaded in 12/2016) [24] and ZINC15 [25] databases (Fig. 2) are analyzed using their PMI profiles. The *merged_dbs* compound set is created by merging all four databases with duplicates removed. Feature interrelations are profiled using the RDKit [33] cheminformatics toolkit and the ChemFP Python library [34, 35]. Compound stereochemistry is removed, compounds are standardized by the IMI eTox standardizer [36] and duplicates are identified using InChIKeys. For each compound, four fingerprints are generated: the PubChemKey (881 bits long) [26], MACCS key (166 bits long) [27] and ECFP4 and ECFP6 fingerprints, both 1024 bits long [28]. To estimate the influence of compound set size on PMI profile, a series of five overlapping ZINC subsets containing 8000, 32,000, 128,000, 512,000 and 2,048,000 randomly selected compounds is prepared.

#### Synthetic accessibility analysis

In this application, ZRFT profiles of several compound sets (Table 1, Fig. 2) with easy (ES) and hard (HS) to synthesize molecules are investigated under the premise that compounds containing feature pairs common in existing molecules are likely to be synthetically accessible.

**Table 1:** Compound sets used in synthetic accessibility assessment

Compound set	Type	Number of compounds
*nonpher*	HS	693,353
*savi*	ES	610,245
*scubidoo*	ES	999,794
*zinc_random*	ES	693,353
*nonpher_complex*	HS	161
*savi_complex*	HS	2930
*scubidoo_complex*	HS	104
*gdb_complex*	HS	3581

ES compounds are easy to synthesize, HS compounds are hard to synthesize. The nonpher compound set corresponds to the S_-_ data set from the SYBA publications [37] in which its construction is described in a detail. savi compounds form the alpha version of the Synthetically Accessible Virtual Inventory (SAVI) Database [38, 39] released on July 2015. scubidoo compounds form the L representative sample of the Screenable Chemical Universe Based on Intuitive Data OrganizatiOn (SCUBIDOO) database [40]. zinc_random compounds are randomly selected from the ZINC15 database [25] and their molecular weight distribution is the same as in the nonpher compound set. The zinc_random compound set corresponds to the S_+_ data set in the SYBA publication [37]. Compounds in _complex sets exceed four complexity thresholds, given by Bertz [41], Whitlock [42], BC [43] and SMCM [44] indices, at once

HS compound set (Additional file 1) is generated by the Nonpher methodology [45]. Nonpher is based on the molecular morphing algorithm [46] in which new structures are constructed by the iterative application of simple structural changes, such as the addition or removal of an atom or a bond. In Nonpher, molecular morphing is stopped when the proposed structure exceeds the threshold [45] of at least one of four monitored complexity metrics (Bertz [41], Whitlock [42], BC [43] and SMCM [44] indices). This procedure was previously optimized [45] to ensure that though generated molecules can be deemed as HS, they are not excessively complex. Nonpher algorithm and compound set construction are described in a detail in the Nonpher and SYBA publications [37, 45].

Three ES compound data sets (Additional file 1) are obtained from the following sources: the Synthetically Accessible Virtual Inventory (SAVI) Database [38, 39], Screenable Chemical Universe Based on Intuitive Data OrganizatiOn (SCUBIDOO) database [40] and ZINC15 database [25]. While the SAVI and SCUBIDOO databases were computationally generated by the application of selected chemical reactions (11 reactions for SAVI and 58 reactions for SCUBIDOO generation) to the given set of chemical building blocks (~ 230,000 building blocks for SAVI and ~ 8000 building blocks for SCUBIDOO generation), the ZINC15 database contains already synthesized commercially available organic compounds. Therefore, compounds in SAVI, SCUBIDOO and ZINC15 databases can be considered as ES. The examples of the *nonpher*, *savi*, *scubidoo* and *random_zinc* compounds are shown in Additional file 2.

Though *savi* and *scubidoo* compound sets are expected to contain only ES compounds, some of these are extremely complex as they exceed all complexity metric (Bertz [41], Whitlock [42], BC [43] and SMCM [44] indices) thresholds [45] at once. Therefore, their *savi_complex* and *scubidoo_complex* subsets containing such extremely compounds are formed (Table 1, Fig. 2, Additional file 1). Because no extremely complex compounds are found in the *zinc_random* set, the additional complex compound set is constructed from the publicly available subset of 50,000,000 molecules from the GDB-17 database [47]. Similarly, extremely complex compounds selected from the *nonpher* compound set form *nonpher_complex* subset. A smaller size of *_complex* compound sets enables their more detailed analysis.

Each compound set is characterized by its ZRFT profile calculated (Eq. 7) against the reference *merged_dbs* compound set using ECFP4 fingerprint 1 024 bits long. ZRFT profiles are compared with the distribution of two fragment based synthetic accessibility measures: SAScore [48] and SYBA [37]. SAScore is calculated by the RDKit toolkit [33] and SYBA by the syba Python package [49].

In addition, following our previous work on synthetic accessibility assessment [37, 45], ZRFT is also applied for the classification of compounds as either ES or HS. ZRFT classification results are compared with random forest (RF) classifier, SAScore and SYBA using the *T*_*MC*_ and *T*_*CP*_ test sets [37] (Additional file 3). The *T*_*MC*_ test set was manually curated from the literature and it consists of 40 HS compounds assessed by experienced medicinal chemists [48, 50–52] and of 40 ES compounds randomly selected from the ZINC15 database [25]. Because small *T*_*MC*_ size may bias results, 30 different *T*_*MC*_ data set instances were generated using the same HS compounds, but different ES compounds [37]. The computationally picked *T*_*CP*_ test set consists of 3 581 excessively complex (i.e., HS) compounds from the GDB-17 database [53] supplemented by 3 581 ES compounds randomly selected from the ZINC15 database [25]. The performance of classification models was assessed by the classification accuracy (*Acc*), sensitivity (*SN*), specificity (*SP*) and area under the ROC curve (*AUC*) calculated for the *T*_*MC*_ and *T*_*CP*_ test sets. For each model, its optimum classification threshold was calculated using the Youden index [54, 55].

SAScore was calculated by the RDKit toolkit [33] and SYBA by the SYBA Python library [49]. The RF classifier was implemented in Scikit-learn [56]. RF model was trained using the training set *S* with compounds encoded by 1024-bits long Morgan fingerprint with radius 2. The training set *S* consists of the *zinc_random* (693 353 ES compounds) and *nonpher* (693 353 HS compounds) compound sets. Two RF hyperparameters were optimized in a grid search: the number of trees (50, 100, 300 and 500) and the maximum number of features considered when looking for the best split (10% out of 1024 = 102, 25% = 256, 50% = 512, 75% = 768, 100% = 1024, $\sqrt{1024}=32$ and ${\mathrm{log}}_{2}\left(1024\right)=10$). The final setting used in this work (100 trees and 32 features) represents the best trade-off between computational efficiency and prediction accuracy [57]. More detailed description of data set construction and of testing methodology is given in the original publication [37].

## Results and discussion

### Chemical database analysis

The number of all and unique standardized compounds in the DrugBank, ChEMBL, PubChem, ZINC and *merged_dbs* compound sets is shown in Table 2 and the overlaps between individual compound sets in Table 3.

**Table 2:** The number of all and unique standardized compounds

	All compounds	Unique compounds
DrugBank	6768	6496
ChEMBL	1,666,863	1,512,302
PubChem	91,221,617	69,081,967
ZINC	285,732,863	157,914,301
*merged_dbs*	378,628,111	213,777,358

Compounds are standardized using IMI eTox standardizer [36] and duplicates are identified using InChIKey calculated after compound standardization

**Table 3:** Overlaps between compound sets

	DrugBank	ChEMBL	PubChem	ZINC
DrugBank	6496	0.307%	0.008%	0.002%
ChEMBL	4647	1,512,302	1.895%	0.279%
PubChem	5854	1,313,209	69,081,967	6.280%
ZINC	3421	443,794	13,412,856	157,914,301

The counts of unique overlapping compounds are shown in the lower triangle, compound set size on the diagonal and the overlap between two compound sets, given as the Jaccard index, in the upper triangle. The Jaccard index J(A, B) between compound sets A and B is calculated as the size of the intersection between A and B divided by the size of the union of A and B: $J(A,B)=\frac{\left|A \cap B\right|}{\left|A \cup B\right|}$

**Fig. 3:**
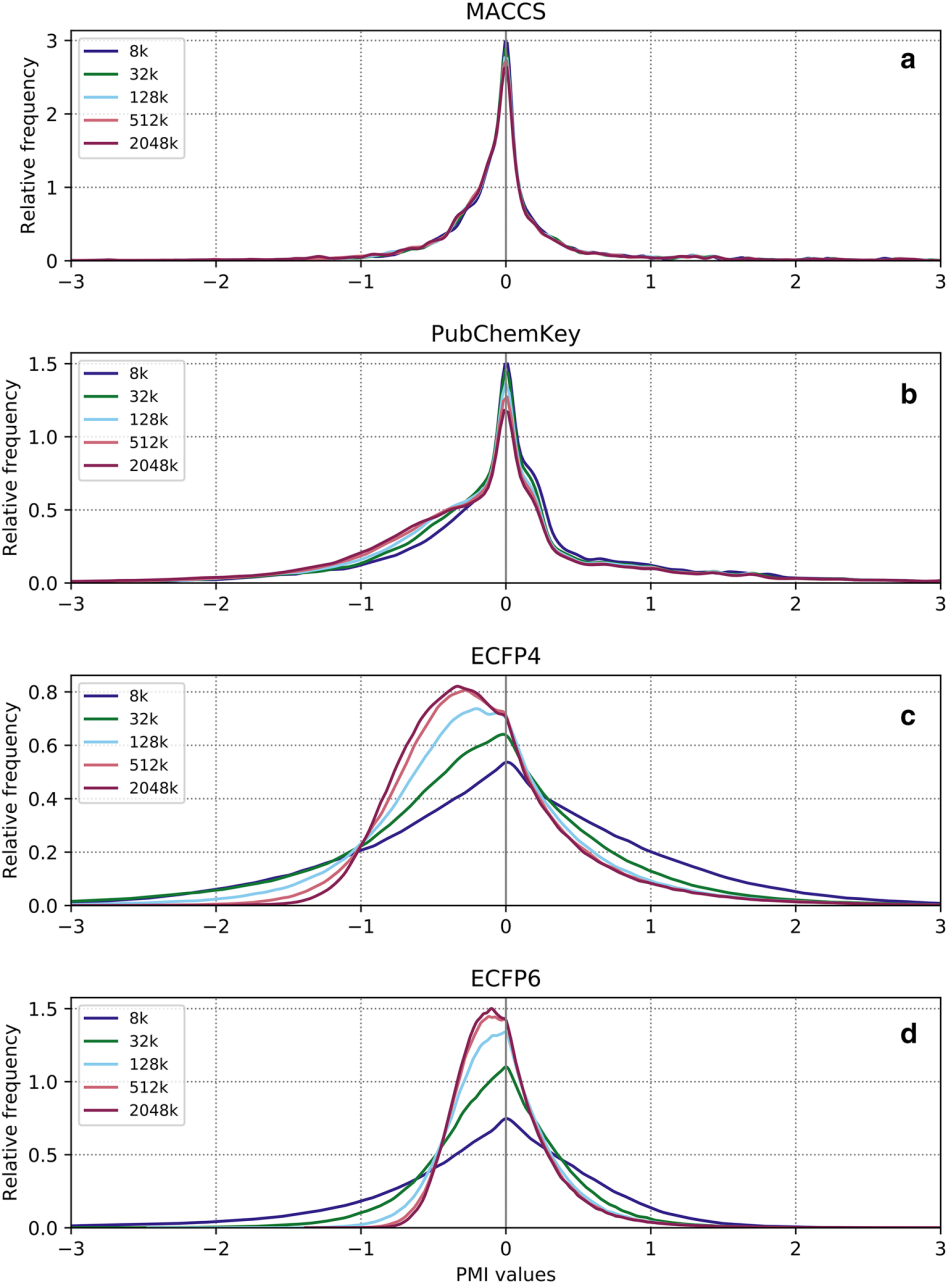
The dependence of PMI profile on compound set size. 5 randomly selected ZINC subsets that contain 8000, 32,000, 128,000, 512,000 and 2,048,000 compounds are profiled using MACCS, PubChemKey, ECFP4 and ECFP6 fingerprints

PMI profiles of increasingly larger randomly selected ZINC subsets are shown in Fig. 3.

With increasing compound set size, MACCS and PubChemKey PMI interrelation profiles are mostly unchanged (Fig. 3a, b) and the overall number of bits set to 1 remains constant (~ 145 out of 168 for MACCS, ~ 645 out of 888 for PubChemKey). In contrast, ECFP interrelation profiles become, with increasing compound set size, more rounded and shifted towards negative PMI values (Fig. 3c, d). Compared to ECFP4, ECFP6 profiles are smoother, because ECFP4 fragment space is a subset of ECFP6 fragment space. Also, ECFP6 profiles shift towards negative values to a lesser extent than ECFP4 profiles (Fig. 3d) meaning that ECFP6 specific interrelations contribute positively.

**Fig. 4:**
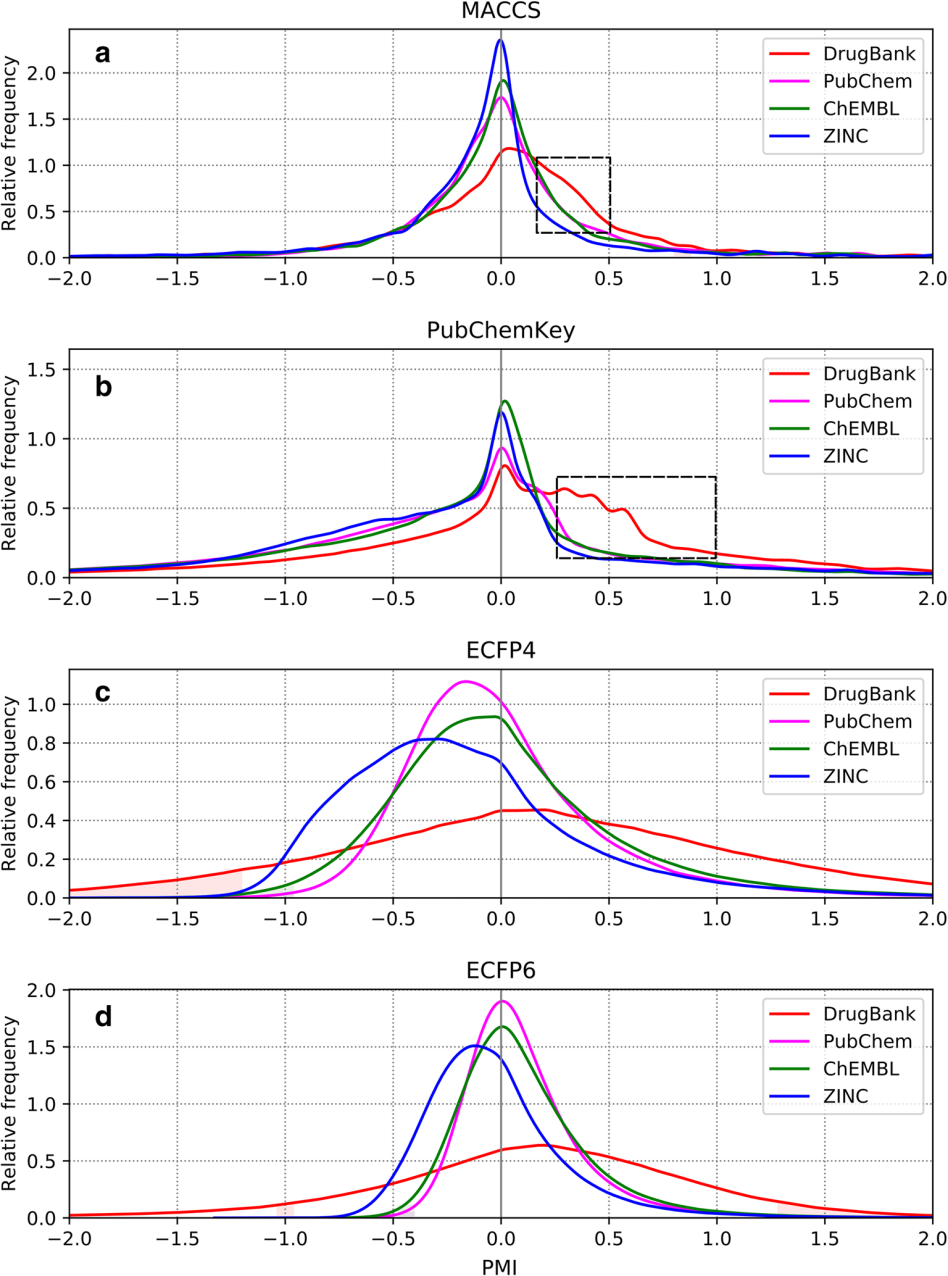
PMI profiles of the DrugBank, PubChem, ChEMBL and ZINC databases using MACCS, PubChemKey, ECFP4 and ECFP6 fingerprints. Dashed rectangles in MACCS and PubChemKey profiles highlight the regions where DrugBank significantly differs from other databases. In this region, 1674 interrelations not present in other databases were identified

The use of MACCS, PubChemKey, ECFP4 and ECFP6 fingerprints for the calculation of PMI profiles of the DrugBank, ChEMBL, PubChem and ZINC databases results in 16 interrelation profiles (Fig. 4).

**Fig. 5:**
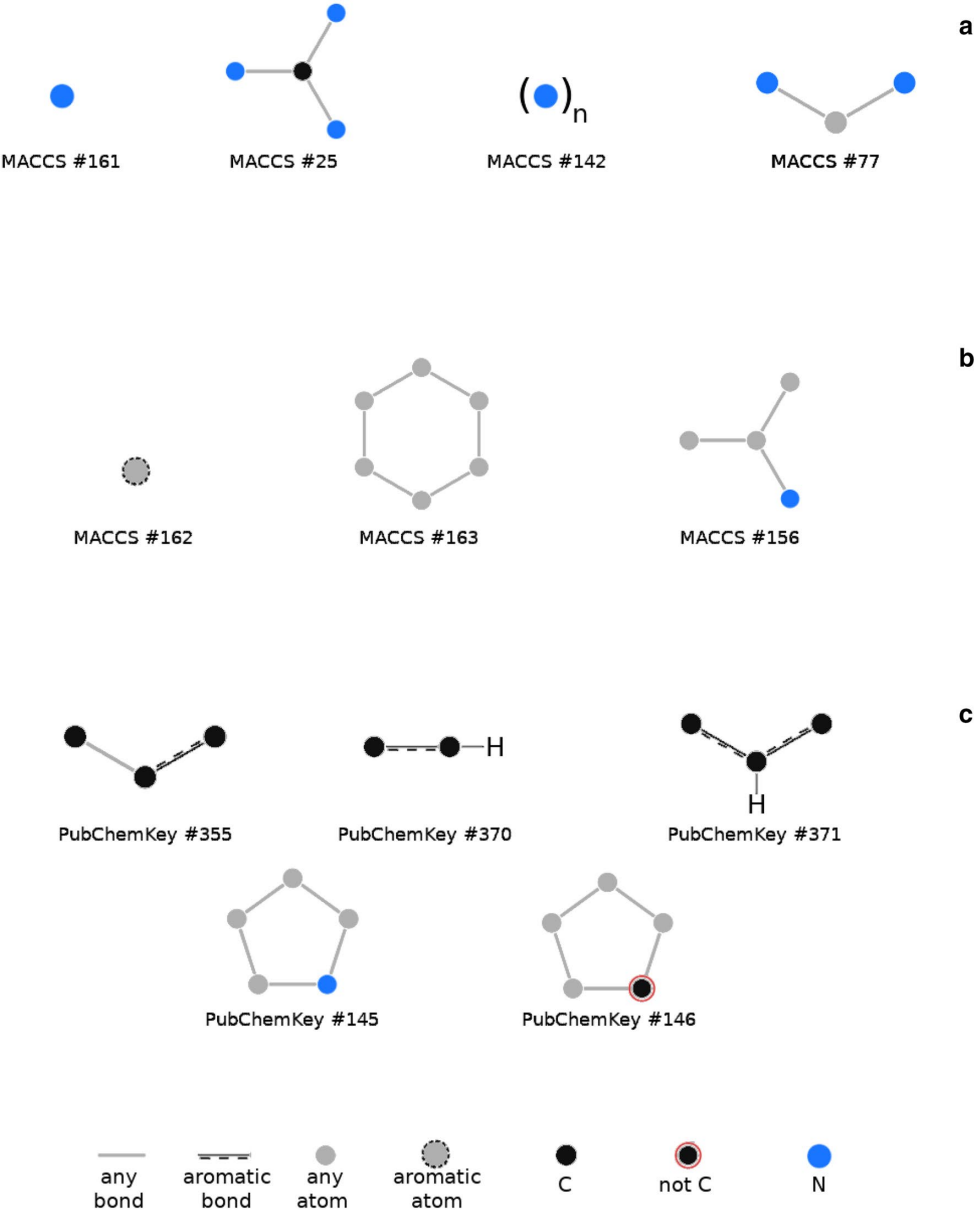
Examples of MACCS and PubChemKey fragments. Generic and specific MACCS fragments (**a**), MACCS (**b**) and PubChemKey (**c**) fragments excusive for DrugBank

PMI profiles derived from MACCS and PubChemKey structural keys peak around zero (Fig. 4a, b). ChEMBL, PubChem and ZINC PMI profiles all show similar negatively skewed distribution indicating that most features are less likely to appear together than separately. In contrast, MACCS and PubChemKey PMI profiles of DrugBank show pronounced right tails indicating the existence of positive interrelations. This is likely due the presence of structural motifs shared within the same classes of drugs. The sharp shape of structural key PMI profiles reflects the fact that fragment dictionaries vary greatly in the scope and overlap. For example, MACCS key defines features as generic as a nitrogen atom (bit #161) alongside features as specific as a methanetriamine substructure (bit #25) (Fig. 5a). Also, some MACCS features imply one another, such as methanetriamine fragment that implies the following features: a nitrogen atom (bit #161), more-than-one-nitrogen atom (bit #142) and nitrogen-any_atom-nitrogen substructure (bit #77) (Fig. 5a).

Still, meaningful conclusions can be drawn from explicitly defined structural features. MACCS PMI range between 0.2 and 0.5 (Fig. 4a), that is more populated in DrugBank compared to other databases, contains 2 306 interrelations with 1 674 being DrugBank exclusive. A majority of these involve various aromatic features (e.g., bit #162), nonaromatic six-membered rings (bit #163) and an NA(A)A pattern (bit #156) (Fig. 5b). Similarly, PubChemKey PMI profile of DrugBank contains, within the range of 0.3 and 1.0 (Fig. 4b), 47 907 interrelations with 36 057 interrelations exclusive to DrugBank. These involve mainly aromaticity-related features (Fig. 5c), such as small substructures with explicit aromatic bonds (e.g., bits #355, #370, #371) and with heteroatoms (bits #145 or #146).

Compared to MACCS and PubChemKey, ECFP interrelation profiles are more regular (Fig. 4c, d) because ECFP fingerprints contain all circular fragments of the given radius. For example, ECFP6 dictionary consists of all possible circular fragments of the radius of 0, 1, 2 and 3 bonds. While PubChem, ZINC and ChEMBL ECFP profiles are negatively skewed, DrugBank ECFP profiles are symmetric and contain more positive PMI values. The flat shape of DrugBank ECFP profile is due to lower DrugBank size (see Fig. 3c and d). The shift of DrugBank ECFP profile to the right is the demonstration of unusual structural properties of drugs that were also described in several previous studies using different methodologies [58–60].

The presence of a higher amount of negative interrelations in ZINC ECFP profile (Fig. 4c, d) means that ZINC contains less co-occurring structural fragments than any other database. This indicates that, in terms of feature interrelations, ZINC contains the most diverse set of compounds. On the other hand, considering that the average database Tanimoto coefficient ${\stackrel{-}{T}}_{C}$ is calculated from 12,497,500 pairwise comparisons generated exhaustively from 5000 compounds, ZINC ${\stackrel{-}{T}}_{C}$ value of 0.14, which is the highest of all databases (Table 4), means that ZINC structures share 14% of ECFP features on average. ZINC can, thus, be considered as the least structurally diverse database. Seemingly contradictory conclusions regarding ZINC diversity are only the manifestation of the fact, that both measures capture different compound properties and reflect, thus, different views of reality. Tanimoto similarity quantifies how are individual features shared between compounds compared to all features present in a compound set *S*. On the other hand, PMI quantifies (Eq. 3) how often features *x* and *y* occur together in the same compound (given by the feature pair co-occurrence probability *p(x, y)*) compared to the chance that they appear in the same compound if they are independent (given as *p(x)·p(y)*). So, if *x* and *y* are present in all compounds in *S*, they positively contribute to pairwise Tanimoto coefficients between structures in *S*. However, their *PMI* will be zero because *p(x,y)* = *1*, *p(x)* = *1*, *p(y)* = *1* and $PMI={\mathrm{log}}_{2}\frac{p(x,y)}{p(x)p(y)}={\mathrm{log}}_{2}1=0$. This means that a compound set can have a high average Tanimoto similarity between the structures and, at the same time, low PMI values. In the case of ZINC compounds, while a high pairwise Tanimoto similarity indicates that they have, out of all studied compound sets, most fragments in common, their low PMI values mean that these fragments are less mutually interrelated.

**Table 4:** Average pairwise Tanimoto similarities ${\stackrel{-}{T}}_{C}$

Compound set	MACCS	PubChemKey	ECFP4	ECFP6
ChEMBL	0.38	0.44	0.12	0.10
DrugBank	0.30	0.32	0.10	0.08
PubChem	0.35	0.40	0.12	0.10
ZINC	0.44	0.45	0.14	0.12

From each compound set, 5000 compounds are selected randomly and all 12,497,500 Tanimoto pairwise similarities ${T}_{C}$ are calculated using MACCS, PubChemKey, ECFP4 and ECFP6 fingerprints, were averaged

### Synthetic accessibility analysis

**Fig. 6:**
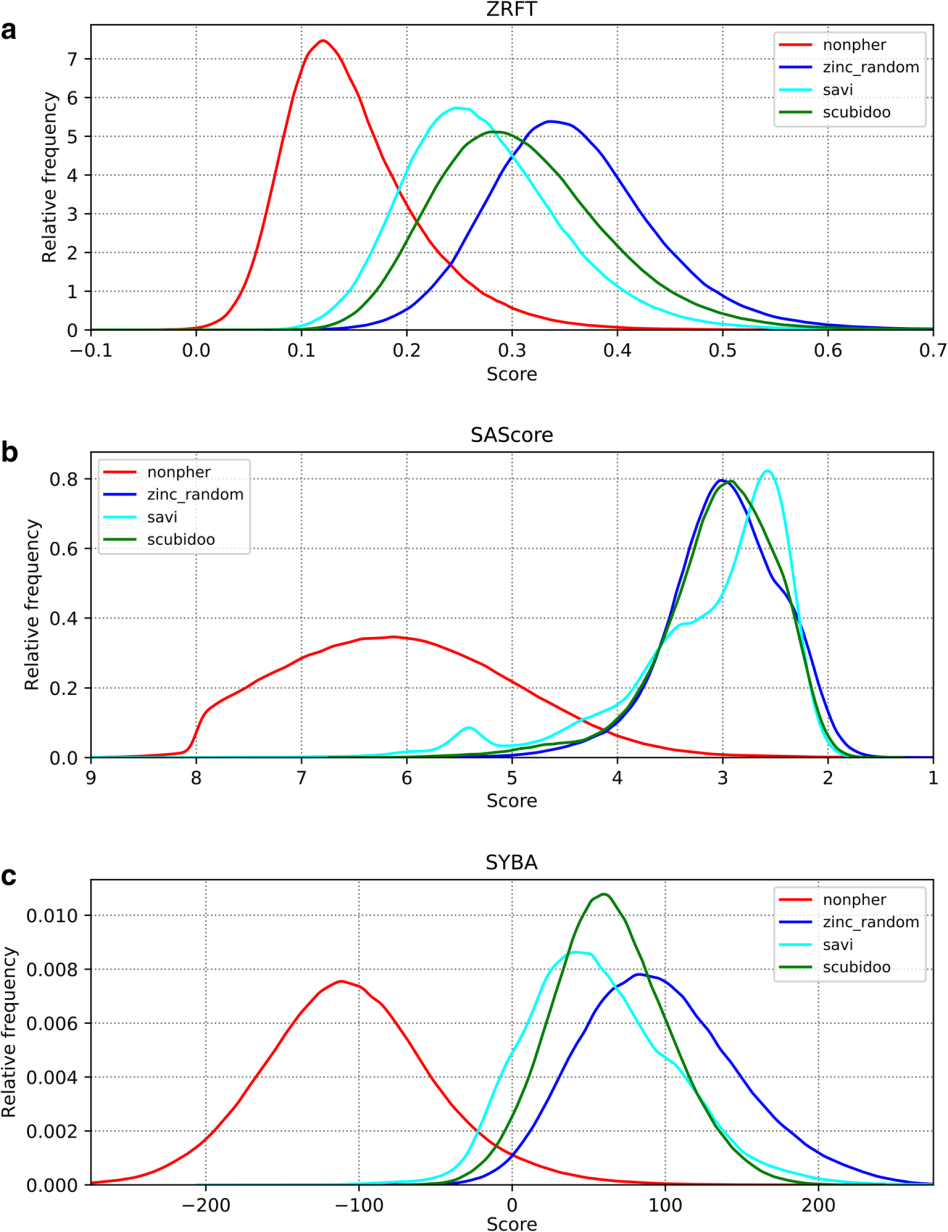
ZRFT profiles and SAScore and SYBA distributions of the *nonpher*, *zinc_random*, *savi* and *scubidoo* compound sets. The *nonpher* compound set contains HS compounds, *zinc_random*, *savi* and *scubidoo* are the compound sets containing ES compounds. ZRFT profiles are calculated using 1024-bits ECFP4 fingerprint with *merged_dbs* as the reference compound set

ZRFT, SAScore and SYBA distributions, of the *nonpher*, *savi*, *scubidoo* and *zinc_random* compound sets are shown in Fig. 6.

While ZRFT profiles (Fig. 6a) and SYBA distributions (Fig. 6c) are smooth, SAScore distributions (Fig. 6b) shows more complex shapes that are likely the result of heuristic complexity penalty used in SAScore calculation [48]. ZRFT profiles (Fig. 6a) show a clear separation between ZINC (i.e., ES) and Nonpher (i.e., HS) [45] compounds. ZRFT values of the computationally generated ES compounds sets, i.e. SAVI and Scubidoo, fall between those of Nonpher and ZINC, closer to ZINC. The same trends can be observed in SYBA and SAScore distributions, albeit SAScore distributions show less distinction between ZINC and SAVI compounds.

**Fig. 7:**
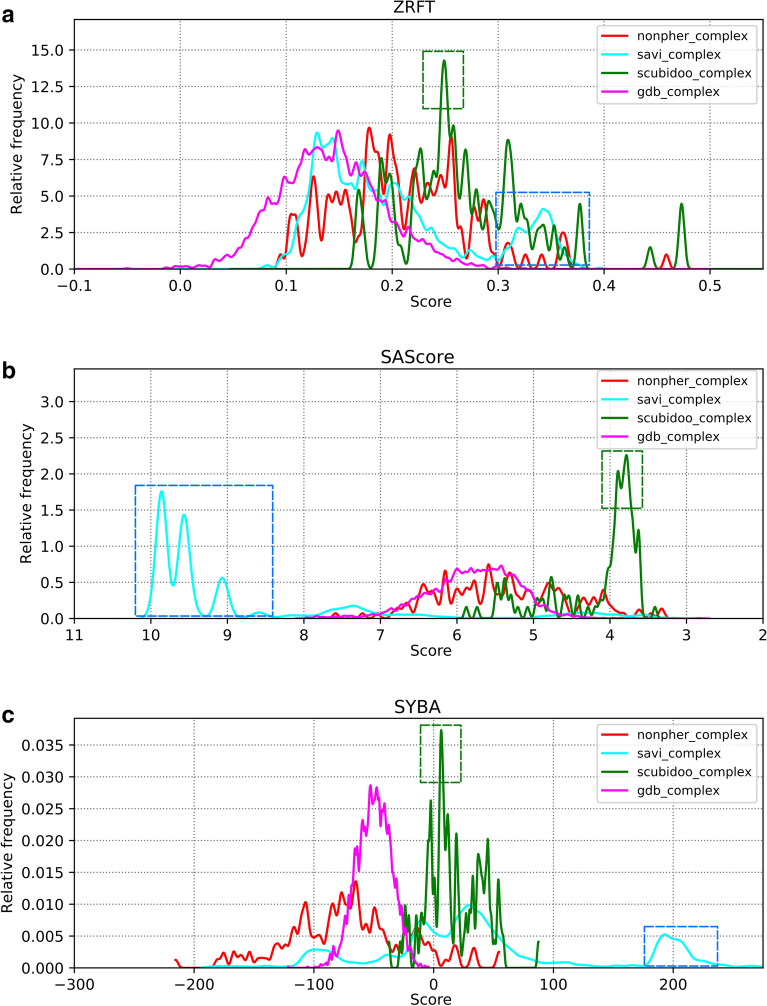
ZRFT profiles and the distribution of SAScore and SYBA of the *nonpher_complex*, *savi_complex*, *scubidoo_complex* and *gdb_complex* compound sets. ZRFT values are calculated using 1024-bits ECFP4 fingerprint with *merged_dbs* as the reference set. Dashed rectangles highlight the regions with interesting SCUBIDOO (green rectangle) and SAVI (blue rectangle) complex compounds

ZRFT profiles and SYBA and SAScore distributions of the *nonpher_complex*, *savi_complex*, *scubidoo_complex* and *gdb_complex* compound sets are shown in Fig. 7.

**Fig. 8:**
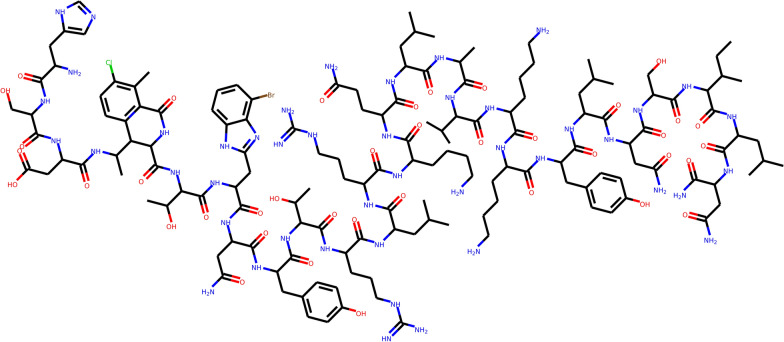
The example of an oligopeptide with ZRFT = 0.35, SAScore = 9.55 and SYBA = 207.54

SYBA, SAScore and ZRFT distributions of the *scubidoo_complex* compound set are shifted toward positive values and contain more values associated with synthetically accessible structures than any other complex compound set. Strong *scubidoo_complex* peaks at ZRFT ~ 0.25 (Fig. 7a), SAScore ~ 3.7 (Fig. 7b) and SYBA ~ 10 (Fig. 7c) are composed mostly by the same 66 structures with five or six membered heterocycles. *savi_complex* compounds are rated differently by all three methods with their SAScore and SYBA distributions being particularly irregular and widespread. Based on their high ZRFT (> 0.3) and SYBA (> 180) values (Fig. 7a, c), 499 SAVI complex compounds should be considered as ES. However, their SAScore higher than 8.5 (Fig. 7b) would rate these compounds as HS. A closer inspection reveals that all these compounds are oligopeptides (Fig. 8) and can be, therefore, synthesized by connecting individual amino acid residues [61]. Because SAScore is designed [48] to assess the SA of drug-like [62] compounds, oligopeptides lie outside its applicability domain. Their structural complexity, incorporated into SAScore using the heuristic complexityScore [48], outweighs individual fragment contributions and contributes unfavorably to their SAScore values. In contrast, both SYBA and ZRFT predict these compounds correctly as ES. Oligopeptides include a large number of fragments that are highly scored because they appear more often in ES than in HS compounds, which is reflected in their high SYBA values. Also, oligopeptides contain ECFP feature pair combinations that fit well within the ZRFT profile of known SA compounds in *merged_dbs*.

**Fig. 9:**
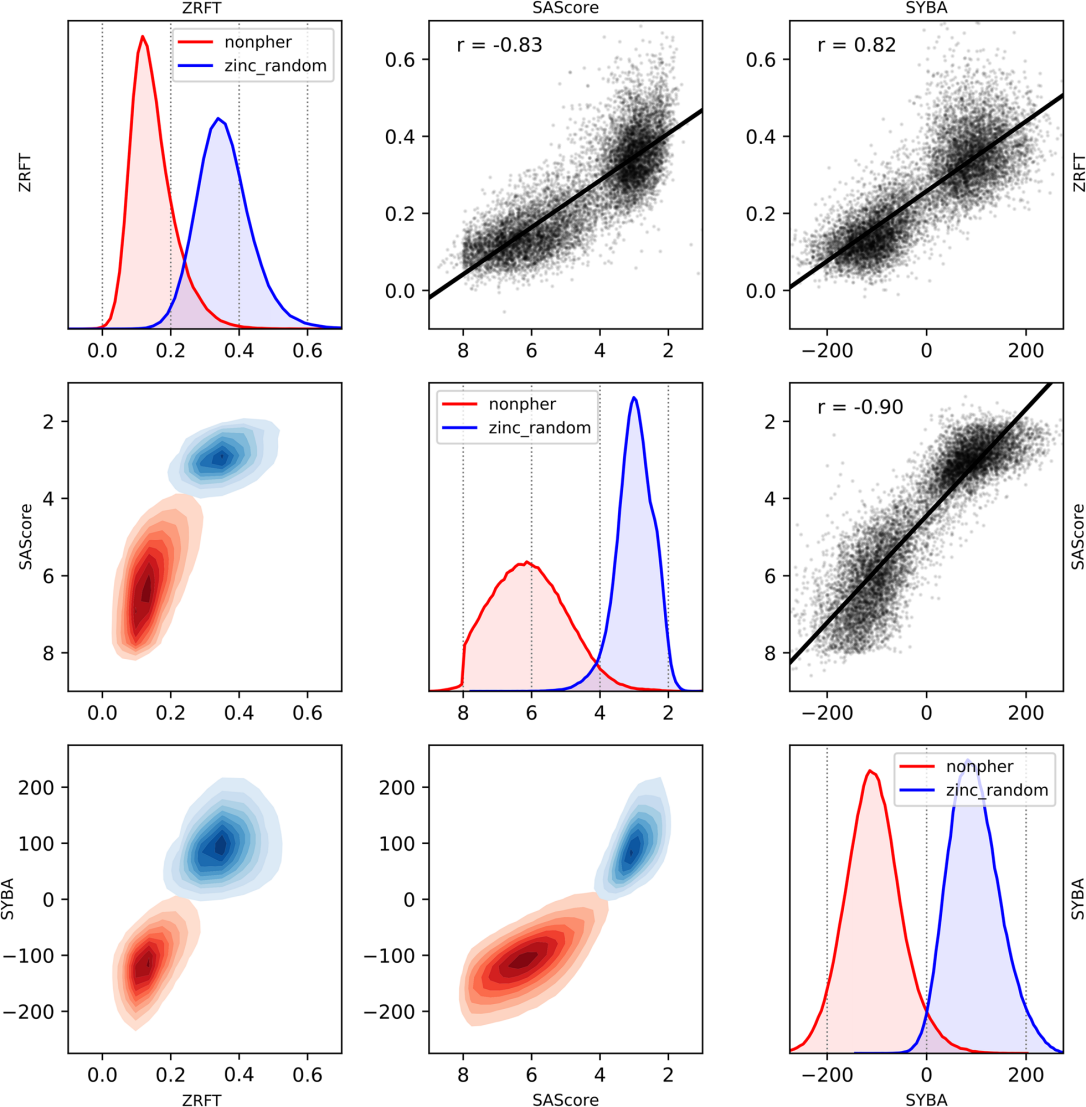
Correlation between ZRFT, SYBA and SAScore. On the diagonal, distributions of individual SA scores for the *nonpher* (i.e., HS) and *zinc_random* (i.e., ES) compound sets are plotted. Above the diagonal, correlations between all SA score pairs are shown. Below the diagonal, pairwise kernel density estimations between all SA score pairs are depicted. Distributions were calculated for 10 000 randomly selected compounds from Nonpher and ZINC databases

The smallest overlap between ZINC and Nonpher compounds and, therefore, the best differentiation between ES and HS compounds was achieved by the SYBA model, followed by SAScore and ZRFT (Fig. 9). ZRFT is strongly correlated (Fig. 9) both with SYBA (*r* = 0.82) and SAScore (*r* = − 0.83) which demonstrates that ZRFT contains a significant amount of information about compound SA.

In addition, the separation between ES and HS compounds in ZRFT density plots (Fig. 9) suggests that ZRFT can be used as a classifier. The comparison between the RF, SYBA, SAScore and ZRFT classification of the T_MC_ and T_CP_ tests sets is given in Tables 5 and 6, respectively.

**Table 5:** The performance of classification models for the manually curated TMC test set

Model	*AUC*	*Acc*	*SN*	*SP*
SYBA	0.903	*0.871*	*0.902*	0.840
SAScore	0.865	0.859	0.799	*0.919*
ZRFT	0.871	0.831	0.827	0.836
RF	0.875	0.842	0.855	0.828

Quality measures *AUC*, *Acc*, *SN* and *SP* are reported as their average values over 30 T_MC_ instances. Results for SYBA, SAScore and RF classification are taken from SYBA publication [37]

**Table 6:** The performance of classification models for the computationally picked T_CP_ test set

Model	*AUC*	*Acc*	*SN*	*SP*
SYBA	0.998	0.988	0.985	0.991
SAScore	*0.999*	*0.990*	*0.986*	*0.993*
ZRFT	0.987	0.945	0.933	0.956
RF	0.995	0.973	0.960	0.986

Results for SYBA, SAScore and RF classification are taken from SYBA publication [37]

Though ZRFT classification is inferior to SYBA, SAScore and RF, its ability to distinguish, using the Youden index optimized threshold of 0.2, between ES and HS compounds is surprisingly high considering that ZRFT is a generic measurement based only on interrelations between structural feature pairs compared to the reference compound set, while SYBA and SAScore are dedicated models trained to estimate compound SA.

## Conclusions

The methodology of pointwise mutual information (PMI) profiling is introduced and its utility is demonstrated for the analysis of structural feature interrelations in publicly available chemical databases and for the analysis and prediction of synthetic accessibility of organic compounds. Interrelation profiles are constructed both from dictionary-based (MACCS and PubChemKey) and hashed circular fragments (ECFP). PMI interrelation profiles of the PubChem, ZINC, ChEMBL and DrugBank databases indicate the presence of both positive and negative feature interrelations. ECFP structural fragments are more suitable for fragment co-occurrence profiling than dictionary-based fragments as they provide more regular interrelation profiles. Unusual favorable fragment combinations of DrugBank compounds manifest themselves by the shift of DrugBank PMI profile to positive values meaning that DrugBank compounds have stronger positive feature interrelations than any other chemical database. Z-standardized relative feature tightness (ZRFT), a PMI-derived measure that quantifies how tightly the query compound set matches the reference compound set, is used to characterize five compound sets with varying degree of synthetic accessibility. Synthetically accessible compounds possess a higher amount of fragment pairs occurring in known molecules. ZRFT profiles are compared with the distributions of SYBA [37] and SAScore [48], two dedicated models for the estimation of synthetic accessibility. In addition, ZRFT is also applied to the classification of compounds as easy (ES) or hard (HS) to synthesize and compared to the results of the random forest (RF), SYBA and SAScore. Though ZRFT classification is inferior to SYBA, SAScore and RF, its ability to distinguish between ES and HS compounds is surprisingly high. Therefore, we may conclude that compound synthetic accessibility is given, to a large extent, by structural feature combinations that can be quantified by ZRFT. However, we would like to stress that ZRFT is not a dedicated measure of synthetic accessibility. Instead, ZRFT is a generic method that only detects interrelations between structural feature pairs and quantifies their match to interrelations in the reference compound set. ZRFT interpretation depends on the context. For example, comparing a compound with the interrelation profile of synthetically accessible compounds will be interpreted differently than comparing it with the interrelation profile of natural products.

For the comparison of chemical databases, PMI interrelation profiles (Eq. 4) are favored over ZPMI profiles (Eq. 5) because Z-score standardization removes information about the absolute PMI values which is usually undesirable for this application. On the other hand, ZRFT is more suitable for the analysis and prediction of compound properties such as synthetic accessibility. While RFT (Eq. 6) captures the strength of interrelations in absolute numbers that can vary widely depending on the reference interrelation profile, ZRFT (Eq. 7) quantifies how well the observed feature pairings match the reference interrelation profile in the units of standard deviation, leading to better interpretability.

The results presented in the current work indicate that structural feature co-occurrence, quantified by PMI or ZRFT profiles, contains a significant amount of information relevant to physico-chemical properties of organic compounds. It must be stressed that neither PMI nor ZRFT are models. PMI is simply the representation of interrelations between feature pairs within a compound set and ZRFT is the measure of a similarity, in terms of feature co-occurrences, between two compound sets (though ZRFT is not a metric as it is not symmetric). This is akin to structural fingerprints, where a fingerprint is the representation of structural features within a compound and the Tanimoto coefficient is the measure of similarity between two fingerprints. The possible use cases of interrelation profiles will be, due to these conceptual parallels, similar to these of binary fingerprints. Consequently, feature interrelation profiles can be potentially used to introduce additional information rich layer to established fingerprint-based methodologies. However, the construction of meaningful interrelation profiles is computationally intensive, which we perceive as one of the biggest limitations of feature interrelation profiling. The study of the influence of the number of compounds on the interrelation profile (Fig. 3) indicates that the number of compounds necessary to yield a meaningful interrelation profile is in the order of 10^5^–10^6^ for ECFP feature vectors. Finally, the interrelation profile is defined by the choice of a feature vector. For an intended use, it may not be straightforward to choose the appropriate feature vector and it may be required to construct a multitude of different interrelation profiles for different feature vectors.

In the future, we plan to further experiment with different types of feature vectors and to adapt the methodology of sparse vectors and matrices in order to decrease computational demands. Furthermore, we will design feature vectors with structural features corresponding to specific functional groups, pharmacophore features etc. with the aim to improve the interpretability of the resulting interrelation profiles. Later, we will also investigate the utility of hybrid feature vectors containing interrelation profiles concatenated with, for example, QAFFP biological fingerprints [63, 64] or with other features of interest. We plan to use interrelation profiling in various cheminformatics applications, such as in biological activity classification or potency prediction, focused chemical library construction, diversity data selection or ensemble modeling using RFT together with domain-specific models for, e.g., natural product likeness assessment [65–67]. Given that interrelation profiles are matrices of numeric values, they can also be used to train machine learning models and to identify and leverage specific feature interrelations that provide most information about the estimated property.

## Supplementary Information

**Additional file 1.** Compound sets used for synthetic accessibility analysis (*nonpher* - 693 353 HS compounds, *zinc_random* – 693 353 ES compounds, *savi* - 610 245 ES compounds, *scubidoo *– 999 749 ES compounds) including excessively complex (i.e., HS) compounds (*nonpher_complex* – 161 compounds, savi_complex – *2 930* compounds, *scubidoo_complex* – 104 compounds, *gdb_complex* – 3 581 compounds).

**Additional file 2.** Structures of randomly selected nonpher, savi, scubidoo and random_zinc compounds.

**Additional file 3.** Compound sets used for synthetic accessibility classification (*T*_*MC*_ and *T*_*CP*_ test sets). Manually curated test set (*T*_*MC*_) consists of 40 HS compounds manually selected from scientific papers and of 30 ES sets, each of them contains 40 compounds randomly selected from the ZINC15 database. Computationally picked test set *T*_*CP*_ consists of 3 581 HS compounds obtained from the GDB-17 database complemented by the same number of compounds randomly selected from the ZINC15 database.

## Data Availability

fip, Python library for interrelation feature profiling is available at https://github.com/lich-uct/fip. fip GitHub repository contains Python code, tutorial in the form of Jupyter notebook and pre-computed CORM matrices of ZINC, PubChem, ChEMBL, DrugBank and merged compound sets. fip is also available as Conda package at https://anaconda.org/LICH/fip.

## Abbreviations

Acc: Accuracy
AUC: Area under the ROC curve
CORM: Co-occurrence relation matrix
COPRM: Co-occurrence probability relation matrix
ES: Easy-to-synthesize
HS: Hard-to-synthesize
MI: Mutual information
PMI: Pointwise mutual information
PMIRM: Pointwise mutual information relation matrix
RF: Random forest
RFT: Relative feature tightness
ROC: Receiver operating characteristic
S: Training set
SA: Synthetic accessibility
SN: Sensitivity
SP: Specificity
SYBA: SYnthetic Bayesian Accessibility
T_CP_: Computationally picked test set
T_MC_: Manually curated test set
ZPMI: Z-standardized pointwise mutual information
ZPMIRM: Z-standardized pointwise mutual information relation matrix
ZRFT: Z-standardized relative feature tightness
