# Supplementary figures and images for: Profiling and analysis of chemical compounds using pointwise mutual information
Source: J Cheminform. 2021 Jan 10;13:3. doi: 10.1186/s13321-020-00483-y (PMC7798221; doi:10.1186/s13321-020-00483-y)

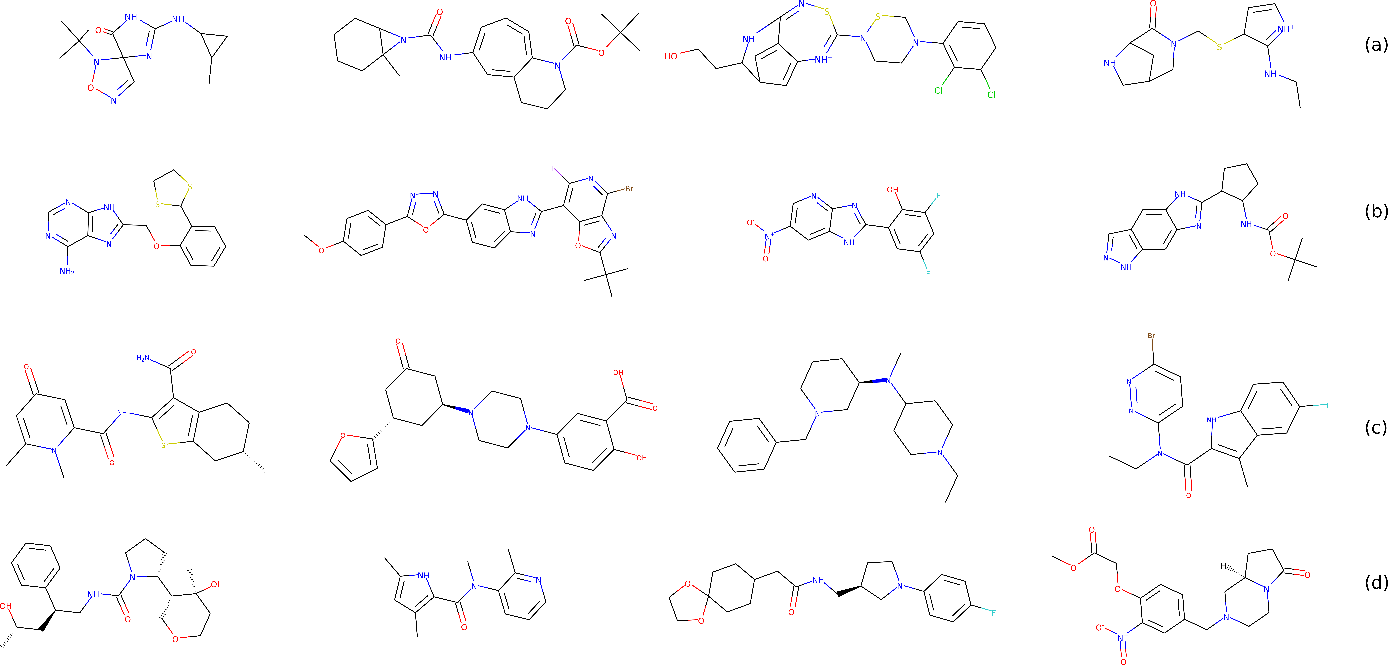


**Figure S1.** The examples of *nonpher* (a), *savi* (b), *scubidoo* (c) and *random_zinc* (d) compounds.

Supplement: Supplementary file 2 — Additional file 2. Structures of randomly selected nonpher, savi, scubidoo and random_zinc compounds. [file 13321_2020_483_MOESM2_ESM.docx]
